# Feasibility and Usability of a Mobile App–Based Interactive Care Plan for Migraine in a Community Neurology Practice: Development and Pilot Implementation Study

**DOI:** 10.2196/48372

**Published:** 2023-10-05

**Authors:** Nathan P Young, Jennifer L Ridgeway, Tufia C Haddad, Sarah B Harper, Lindsey M Philpot, Laura A Christopherson, Samantha M McColley, Sarah A Phillips, Julie K Brown, Kelly S Zimmerman, Jon O Ebbert

**Affiliations:** 1 Department of Neurology Mayo Clinic Rochester, MN United States; 2 Integrated Community Specialty Practice Mayo Clinic Rochester, MN United States; 3 Division of Health Care Delivery Research Mayo Clinic Rochester, MN United States; 4 Department of Oncology Mayo Clinic Rochester, MN United States; 5 Center for Digital Health Mayo Clinic Rochester, MN United States; 6 Community Internal Medicine Mayo Clinic Rochester, MN United States; 7 Qualitative Health Sciences Mayo Clinic Rochester, MN United States; 8 Clinical Informatics and Practice Support Mayo Clinic Rochester, MN United States

**Keywords:** migraine, mobile app, smartphone, care model, feasibility, usability, digital health, remote monitoring, care plan, pilot, mobile health, mHealth, mobile phone, patient-reported outcomes

## Abstract

**Background:**

Migraine is a common and major cause of disability, poor quality of life, and high health care use. Access to evidence-based migraine care is limited and projected to worsen. Novel mobile health app–based tools may effectively deliver migraine patient education to support self-management, facilitate remote monitoring and treatment, and improve access to care. The risk that such an intervention may increase the care team workload is a potential implementation barrier.

**Objective:**

This study aims to describe a novel electronic health record–integrated mobile app–based Migraine Interactive Care Plan (MICP) and evaluate its feasibility, usability, and impact on care teams in a community neurology practice.

**Methods:**

Consecutive enrollees between September 1, 2020, and February 16, 2022, were assessed in a single-arm observational study of usability, defined by 74.3% (127/171) completing ≥1 assigned task. Task response rates, rate and type of care team escalations, and patient-reported outcomes were summarized. Patients were prospectively recruited and randomly assigned to routine care with or without the MICP from September 1, 2020, to September 1, 2021. Feasibility was defined by equal to or fewer downstream face-to-face visits, telephone contacts, and electronic messages in the MICP cohort. The Wilcoxon rank-sum test was used to compare continuous variables, and the chi-square test was used for categorical variables for those with at least 3 months of follow-up.

**Results:**

A total of 171 patients were enrolled, and of these, 127 (74.3%) patients completed ≥1 MICP-assigned task. Mean escalations per patient per month was 0.9 (SD 0.37; range 0-1.7). Patient-confirmed understanding of the educational materials ranged from 26.6% (45/169) to 56.2% (95/169). Initial mean headache days per week was 4.54 (SD 2.06) days and declined to 2.86 (SD 1.87) days at week 26. The percentage of patients reporting favorable satisfaction increased from a baseline of 35% (20/57) to 83% (15/18; response rate of 42/136, 30.9% to 28/68, 41%) over the first 6 months. A total of 121 patients with MICP were compared with 62 patients in the control group. No differences were observed in the rate of telephone contacts or electronic messages. Fewer face-to-face visits were observed in the MICP cohort (13/121, 10.7%) compared with controls (26/62, 42%; *P*<.001).

**Conclusions:**

We describe the successful implementation of an electronic health record–integrated mobile app–based care plan for migraine in a community neurology practice. We observed fewer downstream face-to-face visits without increasing telephone calls, medication refills, or electronic messages. Our findings suggest that the MICP has the potential to improve patient access without increasing care team workload and the need for patient input from diverse populations to improve and sustain patient engagement. Additional studies are needed to assess its impact in primary care.

## Introduction

### Background

Approximately 36 million people in the United States, 21% female and 10.7% male, are affected by migraine or severe headache, with the highest prevalence between the ages of 18 and 44 years [1,2]. Migraine is a major cause of disability and poor quality of life, especially in women of childbearing age and historically disadvantaged populations [1].

Migraine has a significant impact on health care use [3,4]. In 2016, migraine care was delivered in 4 million emergency department visits and 4.3 million office visits [5]. Migraine care is predominately managed by primary care providers (PCPs) and headache specialists, but limited access to health care professionals is exacerbated by staffing shortages in both groups [6-8]. Substantial variation exists in how providers assess and care for migraine [9], introducing inefficiencies and quality of care variability [10]. Patients with chronic migraine, in particular, report high levels of disability and low satisfaction with care [11-14]. Effective evidence-based acute and preventive migraine therapies exist but remain underused [10,15-17], especially for patients with often underdiagnosed chronic migraine [13,16,18].

App-based mobile health (mHealth) remote monitoring for migraine has been demonstrated to be a promising intervention for standardizing the assessment and monitoring of migraine [19]. App-based mHealth remote monitoring may facilitate the delivery of migraine education and behavioral treatments [20] to improve self-management, streamline health care team communications, and reduce health care use. mHealth apps integrated with the electronic health record (EHR) [21] have the potential to provide secure and easily accessible patient-reported data and outcomes for clinical decision-making [22]. However, the type and volume of data stored in the EHR may unnecessarily burden downstream work for migraine care teams without positively affecting patient outcomes [23]. EHR-integrated mobile app–based mHealth tools that organize and present actionable data to health care teams need to be developed and evaluated in clinical practice.

### Objectives

Our team developed a novel, EHR- and mobile app–integrated Migraine Interactive Care Plan (MICP) and implemented it in a community neurology practice. The aims of this multimethod pilot study were to evaluate (1) the feasibility and usability of the MICP, (2) downstream patient health care use, and (3) the impact on care team workload. This study was designed to provide opportunities for MICP design before scaling the effort for future investigation.

## Methods

### Practice Setting

This investigation was conducted in a community general neurology practice at the Mayo Clinic, Rochester, Minnesota [24]. The practice uses a collaborative model in which the neurologists support PCPs with electronic and curbside consultations. Neurologists also conduct traditional face-to-face and telemedicine visits with patients referred from primary care. At the time of a face-to-face consultation, the neurologist recommends a migraine treatment plan that is implemented and managed by a neurology care team. The neurology care team communicates with patients via telephone or secure electronic messaging to monitor and provide ongoing care. Follow-up visits may be conducted with the neurology care team to optimize the plan or the PCP for the ongoing monitoring of stable patients.

### MICP Development

Care Plans are designed using the existing EHR functionality (Epic Systems Corporation; Epic MyChart Care Companion module) and delivered through a mobile app on the patient’s smartphone or tablet. Patients submit symptom assessments and physiologic data through the app, and the results are available in the EHR and through care team dashboards. If patient-generated health data fall outside of predetermined parameters, patients receive education to facilitate self-management, and care may be escalated via direct messages to the managing care team.

The MICP was developed between February 2019 and August 2020 and was led by an investigator from the community neurology practice (NPY) and an implementation coordinator from the Mayo Clinic Center for Digital Health with training and expertise in the user-centered design of digital applications. The design approach was iterative and engaged representatives of headache neurology, family medicine, internal medicine, and nursing. The collaboration also included a clinical nurse specialist, an informatics specialist, business analysts, product specialists, health system engineers, and IT programmers or analysts.

The development team agreed upon the following guiding principles and goals during the MICP design process. The expert consensus recommendations to improve medical communication in migraine management [9] were reviewed and served as a primary guideline with a focus on the assessment of headache frequency, treatment frequency, and function. The MICP was designed with full integration into the existing Mayo Clinic mobile app and the EHR. Existing Epic EHR functionality including flow sheets and questionnaires were used. The MICP was intended for broad use in patients with the full spectrum of migraine, including episodic and chronic migraine, and patients cared for by both PCPs and headache specialists. We aimed to minimize the collection of patient-reported data that were not anticipated to be valued by patients or needed by the care team to recommend or modify a migraine treatment plan. We aimed to collect and display clinic data in a user-friendly manner for both patients and provider care teams. We aimed to use the MICP as a platform to revise the existing Mayo Clinic migraine patient education content using a smartphone-friendly display. We intended to design the MICP in a way that would minimize or reduce the downstream work required by the migraine care team by incorporating education and self-management advice for common patient concerns, leading to telephone calls or electronic messages to the care team. The development team did not include patients with migraine.

### MICP Components

#### Assessment Tools

Patients enrolled in the MICP for a renewable 3-month period, during which they received assessments of health status and satisfaction with the treatment plan. Each week, the patients were assigned to complete a *Migraine Check-In* assessment of headache frequency and acute treatment frequency impact on absenteeism, reduced productivity, and reduced joy in daily activities. The *Migraine Check-In* was developed by our team using expert consensus guidelines [9] and input from health content editors and writers. Each week, patients were also assigned to complete a *Medication Check-In* of medication concerns. Each month, patients assessed their satisfaction with their current migraine treatment plan on a 5-point Likert scale. At baseline and every 3 months, patients completed the Migraine Disability Assessment Score (MIDAS) to assess migraine-related disability [25]. A summary of the assessment tools and workflow is provided in Multimedia Appendix 1.

#### Data Display

Patient-entered data were viewable by the care team within the Epic Synopsis section of the EHR. All numeric data were viewable by clinicians in a color-coded visual graph of the responses over time. A dashboard of patients enrolled in the MICP was built to allow the care team to centralize the viewing of the population enrollment. The dashboard included filters to allow the identification of patients. For example, a filter could be applied to identify patients based on their engagement in the MICP or their last reported satisfaction or level of disability. The patients were also able to view their responses in the MICP app.

#### Education Content

**Figure 1:**
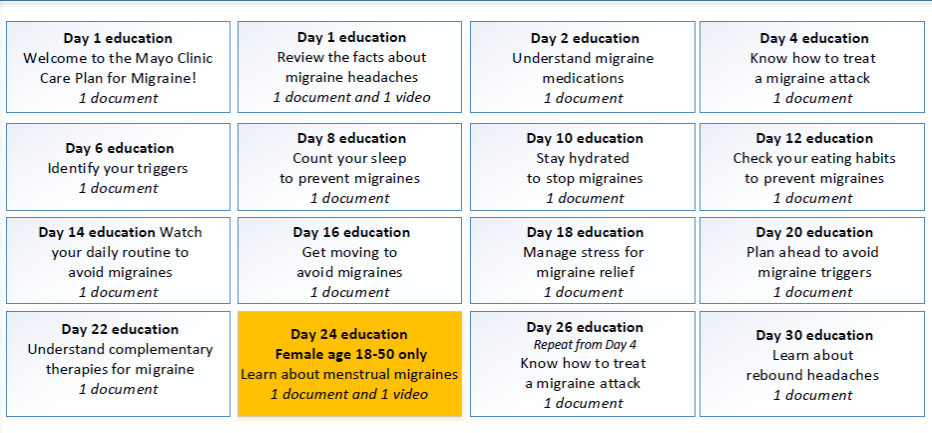
Summary of the Migraine Interactive Care Plan education content and delivery schedule.

The existing Mayo Clinic migraine educational materials were modified by the study team for mobile-friendly viewing. Migraine education content was pushed based on a predetermined schedule over the course of the first month and was viewable within an “Education Library,” which could be accessed on demand. Education on the topic of rebound headaches was provided to patients if they answered “yes” to an MICP question regarding their concerns about rebound headaches. A summary of the educational materials and content delivery schedule is shown in Figure 1.

#### Embedded Logic and Care Escalations

The MICP was designed to include embedded logic based on a patient response to an assessment that would trigger an “Escalation” such as just-in-time education, direction to contact the migraine care team, or an electronic message to the care team. The MICP escalations are listed in Table 1.

**Table 1:** Summary of the Migraine Interactive Care Plan care team escalations.

Escalation name		Frequency	Escalation outcome
**Clinical question**			
	Medication refill	Weekly medication check-in questionnaire	Electronic message to care team
	Medication questions	Weekly medication check-in questionnaire	Electronic message to care team
	Medication concern	Weekly medication check-in questionnaire	Electronic message to care team
	Medication cost concerns	Weekly medication check-in questionnaire	Electronic message to care team
	Worried about a rebound headache	Weekly medication check-in questionnaire	Electronic message to care team and pushed education
	Patient dissatisfied with the migraine care plan	Monthly questionnaire	Electronic message to care team if somewhat or very dissatisfied
	Hospital admission	Triggered by hospital admission	Electronic message to care team
	Hospital discharge	Triggered by hospital dismissal	Electronic message to care team
**Patient noncompliance**			
	Patient not engaging	Triggered by patient inactivity after 1 wk	Electronic message to care team
	Re-enrollment request	Patient is asked if they want to continue using the care plan on day 89 of their care plan journey	Electronic message to care team
	Discontinue patient request	Patient is asked if they want to continue using the care plan on day 89 of their care plan journey	Electronic message to care team

### Pilot Study Participants

The MICP was evaluated in the Mayo Clinic community practice (Rochester, Minnesota). Eligible patients were referred by PCPs to the neurologist for evaluation and treatment of headache as part of the usual practice. An electronic order granting access to the MICP was entered by the neurologist during the clinical encounter. All patients enrolled in the MICP were diagnosed with migraine of any subtype [26] by a neurologist, were aged ≥18 years, had an established Patient Web-Based Services account, and reported owning a smartphone (iOS 10 and higher; Android OS 5.0 and higher). Patients were excluded if they were aged <18 years; had a reading level in English less than eighth grade; were pregnant or lactating; or had dementia, cognitive impairment, or physical condition that limits the ability to use a mobile device; had an uncontrolled mental illness; or had active drug or alcohol abuse. Patients residing in a skilled nursing facility, enrolled in hospice care, identified as end-of-life care, and enrolled in another care plan were also excluded.

### Pilot Study Design

#### MICP Usability and Clinical Outcomes

All consecutive MICP enrollees between September 1, 2020, and February 16, 2022, were assessed in an observational study. Usability and patient-reported clinical outcomes were assessed through patient surveys in the MICP and metrics generated by app use. Usability was defined as 74.9% (128/171) of enrolled patients completing at least 1 MICP task. The assessment included the number, type, and rate per month of care team escalations and the percentage of patients responding “I understand” after viewing migraine educational materials. In addition, we observed the percentage of patients responding to weekly headache tracking reminders, medication check-ins, monthly patient satisfaction surveys, and MIDAS completion at 3 and 6 months. Outcomes of individual patient-reported clinical outcomes from the weekly *Migraine Check-In* (including headache days, treatment days, missed work or school days, reduced productivity days, and reduced joy days) MIDAS total score and patient satisfaction were observed over time and summarized. Response rates for individual assigned tasks were defined by the percentage of assigned tasks that were completed by the patients and were also summarized.

#### MICP Feasibility and Health Care Use

For the first year after implementation, all eligible patients were prospectively recruited and randomly assigned to routine migraine management with or without the MICP. The randomization schedule was reviewed by an unblinded neurologist at the time of each consecutive clinical visit for the primary diagnosis of migraine. Patients randomized to receive the MICP were informed during the clinical visit when the neurologist entered an order granting patient access to the MICP. All MICP users enrolled between September 1, 2020, and February 16, 2022, with at least 3 months of clinical follow-up were compared with a control group that was identified over the first year from September 1, 2020, to September 1, 2021.

The feasibility of the MICP was defined by equal to or fewer downstream clinic visits, telephone contacts, and electronic messages managed by the neurology care team.

For both study groups, the neurologist recommended, discussed, and documented a migraine treatment plan as part of usual practice. The neurologist’s clinical note was viewable by both groups via the Mayo Clinic mobile app or web-based patient portal access. All patients were instructed to contact the treatment care team in the usual fashion using the telephone or electronic messaging and to attend follow-up visits when recommended. Outcome comparisons between the intervention and control groups were not possible because the control group receiving usual care did not receive the same standardized patient-reported outcome assessments.

Demographic data and the occurrence of face-to-face visits, telephone contacts, electronic messages, and emergency department visits were extracted from the EHR for both cohorts.

### Ethical Considerations

This study was deemed nonhuman subjects research by the Mayo Clinic Institutional Review Board (#20-008772), and the requirement for written informed consent was waived.

### Data Analysis

As this was a feasibility study, power calculation was not performed. Patients with <3 months of follow-up were excluded. Descriptive statistics were reported as mean (SD) for continuous variables and as frequencies and percentages for categorical variables. The Wilcoxon rank-sum test was used to compare continuous variables. The chi-square test was used to compare categorical variables. Analysis was performed using SAS (version 9.4; SAS Institute). All tests were 2-sided, and *P* values <.05 were considered statistically significant.

## Results

### Demographic Characteristics

A total of 171 patients were enrolled in the MICP. The demographics of all the MICP users are summarized in Table 2. The mean age of the MICP users was 42 (SD 11.7; range 21-75) years. Most patients were female (155/171, 90.6%), non-Hispanic and non-Latinx (167/171, 97.7%), White (161/171, 94.2%), married (83/171, 48.5%), and English speaking (170/171, 99.4%). The mean number of days of enrollment was 132 (SD 99), with 33.3% (57/171) of patients choosing to re-enroll after 3 months, 7.6% (13/171) choosing to discontinue, and 58.5% (100/171) not responding to the re-enrollment questionnaire.

**Table 2:** Demographic characteristics of the Migraine Interactive Care Plan users (N=171).

Demographic		Values
Age (years), mean (SD; range)		42 (11.7; 21-75)
**Age group (years), n (%)**		
	18-34	56 (32.7)
	35-49	75 (43.9)
	50-64	33 (19.3)
	65-75	6 (3.5)
**Sex, n (%)**		
	Female	155 (90.6)
	Male	16 (9.4)
**Marital status, n (%)**		
	Married	83 (48.5)
	Single	62 (36.3)
	Divorced	19 (11.1)
	Life partner	3 (1.8)
	Separated	3 (1.8)
	Widowed	1 (0.6)
**Ethnicity, n (%)**		
	Hispanic or Latinx	1 (0.6)
	Mexican	2 (1.2)
	Non-Hispanic and non-Latinx	167 (97.7)
	Choose not to disclose	1 (0.6)
**Race, n (%)**		
	African American or Black	5 (2.9)
	Asian	3 (1.8)
	White	161 (94.2)
	Choose not to disclose	2 (1.2)
**First language, n (%)**		
	English	170 (99.4)
	Somali	1 (0.6)

### MICP Usability

The MICP overall task completion rates are summarized in Table 3. Of the 171 patients, 127 (74.3%) completed at least 1 task assigned by the MICP. Only 17% (29/171) patients completed >50% of the assigned tasks.

**Table 3:** Migraine Interactive Care Plan task completion rates (N=171).

Completion rate (%)	Patients, n (%)
0	44 (25.7)
1-10	33 (19.3)
11-20	20 (11.7)
21-30	19 (11.1)
31-40	11 (6.4)
41-50	15 (8.8)
51-60	6 (3.5)
61-70	5 (2.9)
71-80	7 (4.1)
81-90	6 (3.5)
91-100	5 (2.9)

### MICP Care Team Escalations

The care team escalations and frequencies are summarized in Table 4. The most common escalations were related to patient not engaging with the MICP (354/738, 48%). The overall average number of escalations per patient per month was 0.9 (SD 0.37; range 0.0-1.7) escalations.

**Table 4:** Frequency and average monthly rate per patient of the migraine care team escalations.

Escalations		2022					2023										
		Sep	Oct	Nov	Dec	Jan		Feb	Mar	Apr	May	Jun	Jul	Aug	Sep	Oct	Nov
**Clinical question, n (%)**																	
	Medication refill (n=49)	0 (0)	5 (10.2)	0 (0)	2 (4.1)	1 (2)		11 (22.4)	3 (6.1)	5 (10.2)	4 (8.2)	4 (8.2)	3 (6.1)	5 (10.2)	1 (2)	1 (2)	3 (6.1)
	Medication questions (n=19)	0 (0)	1 (5.3)	0 (0)	3 (15.8)	2 (10.5)		4 (21.1)	0 (0)	1 (5.3)	0 (0)	0 (0)	2 (10.5)	3 (15.8)	0 (0)	1 (5.3)	2 (10.5)
	Medication concern (n=41)	0 (0)	2 (4.9)	4 (9.8)	3 (7.3)	4 (9.8)		2 (4.9)	7 (17.1)	3 (7.3)	1 (2.4)	2 (4.9)	1 (2.4)	4 (9.8)	2 (4.9)	5 (12.2)	1 (2.4)
	Admission (n=11)	0 (0)	0 (0)	0 (0)	1 (9.1)	1 (9.1)		1 (9.1)	1 (9.1)	3 (27.2)	0 (0)	0 (0)	2 (18.2)	0 (0)	1 (9.1)	0 (0)	1 (9.1)
	Discharge (n=43)	0 (0)	0 (0)	0 (0)	4 (9.3)	4 (9.3)		3 (7)	4 (9.3)	5 (11.6)	3 (7)	1 (2.3)	3 (7)	2 (4.7)	6 (14)	4 (9.3)	4 (9.3)
**Patient noncompliance, n (%)**																	
	Patient not engaging (n=354)	0 (0)	0 (0)	0 (0)	16 (4.5)	40 (11.3)		41 (11.6)	62 (17.5)	33 (9.3)	23 (6.5)	13 (3.7)	27 (7.6)	20 (5.6)	25 (7.1)	30 (8.5)	24 (6.8)
	Patient dissatisfied with care plan (n=14)	0 (0)	1 (7.1)	1 (7.1)	1 (7.1)	1 (7.1)		0 (0)	0 (0)	0 (0)	1 (7.1)	1 (7.1)	1 (7.1)	3 (21.4)	1 (7.1)	0 (0)	3 (21.4)
	Re-enrollment request (response=yes; n=58)	0 (0)	0 (0)	3 (5.2)	9 (15.5)	3 (5.2)		2 (3.4)	10 (17.2)	5 (8.6)	2 (3.4)	9 (15.5)	3 (5.2)	1 (1.7)	4 (6.9)	3 (5.2)	4 (6.9)
	Re-enrollment request (response=no; n=18)	0 (0)	0 (0)	0 (0)	0 (0)	0 (0)		1 (5.6)	3 (16.7)	0 (0)	1 (5.6)	4 (22.2)	0 (0)	1 (5.6)	3 (16.7)	4 (22.2)	1 (5.6)
	Re-enrollment no response (n=117)	0 (0)	0 (0)	2 (1.7)	17 (14.5)	3 (2.6)		4 (3.4)	27 (23.1)	10 (8.5)	12 (10.3)	17 (14.5)	7 (6)	4 (3.4)	9 (7.7)	2 (1.7)	3 (2.6)
**Billing questions, n (%)**																	
	Billing concerns (n=14)	1 (7.1)	2 (14.3)	1 (7.1)	2 (14.3)	2 (14.3)		1 (7.1)	0 (0)	1 (7.1)	0 (0)	2 (14.3)	0 (0)	0 (0)	0 (0)	0 (0)	2 (14.3)
Total (n=738), n (%)		1 (0.1)	11 (1.5)	11 (1.5)	58 (7.9)	61 (8.3)		70 (9.5)	118 (156)	67 (9.1)	47 (6.4)	55 (7.5)	48 (6.5)	40 (5.4)	53 (7.2)	49 (6.6)	49 (6.6)
Monthly census, n		29	34	35	65	66		72	84	64	60	51	38	39	43	44	46

### MICP Education Task Completion Rate

**Figure 2:**
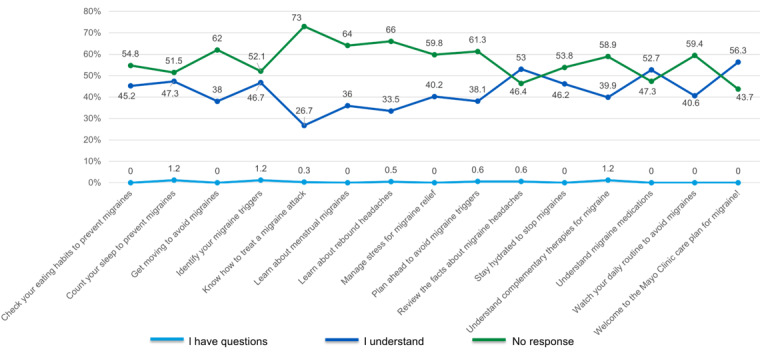
Migraine Interactive Care Plan patient education content response rates.

The education task response rates are summarized in Figure 2. After reviewing the educational materials, the percentage of patients confirming “I understand” ranged from 26.7% (89/333) to 56.3% (94/167). The highest rate of engagement was observed for the first 3 scheduled education tasks, with more than half of the patients indicating “I understand” for the first 3 consecutive scheduled education content titled “Welcome to the Mayo Clinic care Plan for Migraine” (94/167, 56.3%), “Review the Facts About Migraine Headaches” (88/166, 53%), and “Understand Migraine Medications” (89/169, 52.7%). The educational materials with the least confirmation were delivered between 24 and 30 days after enrollment and included “Know How to Treat a Migraine Attack” (89/333, 26.7%), “Learn About Rebound Headaches” (68/203, 33.5%), and “Learn About Menstrual Migraine” (64/178, 36% of women).

### Patient-Reported Migraine Outcomes and Response Rates

**Figure 3:**
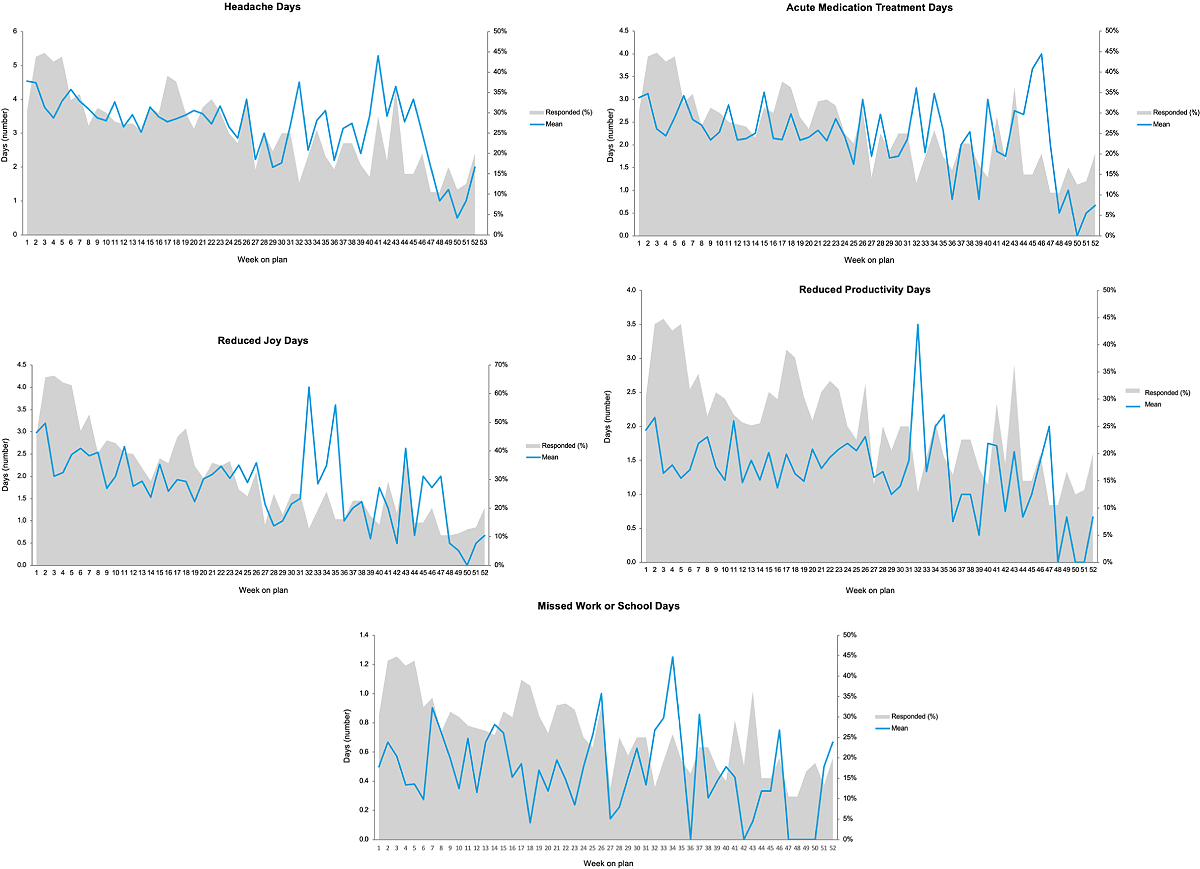
Migraine Interactive Care Plan patient-reported outcomes and weekly response rates.

Figure 3 summarizes patient-reported migraine outcomes and response rates to individual MICP-assigned tasks. Response rates are reported as the percentage of patients completing an MICP-assigned task.

#### Total Headache Days Per Week

The mean weekly reported headache days at enrollment were 4.54 (SD 2.06) days and trended down to 2.86 (SD 1.87) days at week 26. The mean weekly headache days from baseline to 26 weeks was 3.71 (SD 2.27; range 2.86-4.54) days. From week 27 to 52, the mean was 3.05 (SD 2.25; range 2.00-5.29) days. The initial response rate was 29.8% (51/171). The response rate increased to a peak of 45% (77/171) at week 3 and remained constant throughout week 5. After week 5, the response rate decreased. The mean weekly response rates from baseline to week 26 were 32% (SD 7%) and 17.4% (SD 6%) for the remainder of the year.

#### Headache Treatment Days Per Week

The mean weekly headache treatment days at enrollment was 3.04 (SD 2.09). Mean weekly treatment days from baseline to 26 weeks was 2.50 (SD 1.97; range 2.09-3.15) days over the first 26 weeks. The mean treatment days ranged from 0 to 4. From week 26 to 52, the mean weekly treatment days was 2.11 (SD 2.12; range 0-4.0). The response rate peaked at 45% (77/171) and remained highest through week 5, followed by a downward trend.

#### Missed Work or School Days Per Week

Mean missed work or school days per week at baseline was 0.5 (SD 1.20) days. From baseline to week 26, the mean was 0.53 (SD 1.26) days, and from week 27 to 52, the mean was 0.46 (SD 0.95) days. The response rate was 25.7% (44/171) initially and varied between 11.1% (19/171) and 45% (77/171) over 52 weeks, without a consistent trend.

#### Reduced Joy Days Per Week

Mean reduced joy days at enrollment was 2.98 (SD 2.1) days and 2.25 (SD 2.16; range 1.43-3.19) days from baseline to week 26. The initial response rate was 46.2% (79/171), peaked at 66.1% (113/171), and remained highest through week 5. The mean response rate from baseline to week 26 was 41% (SD 13%; range 39%-66%). From week 27 to 52, the mean response rate was 19% (SD 6%).

#### Reduced Productivity Days Per Week

Mean reduced productivity days at baseline was 1.95 (SD 1.77) days and 1.54 (SD 1.85; range 1.17-2.13) days from baseline to week 26. The initial response rate was 29.8% (51/171), peaked at 45% (77/171), and remained highest through week 4. The mean response rate from baseline to week 26 was 31% (SD 7%; range 23%-45%). From week 27 to 52, the mean response rate was 19% (SD 6%).

#### MIDAS Questionnaire

**Figure 4:**
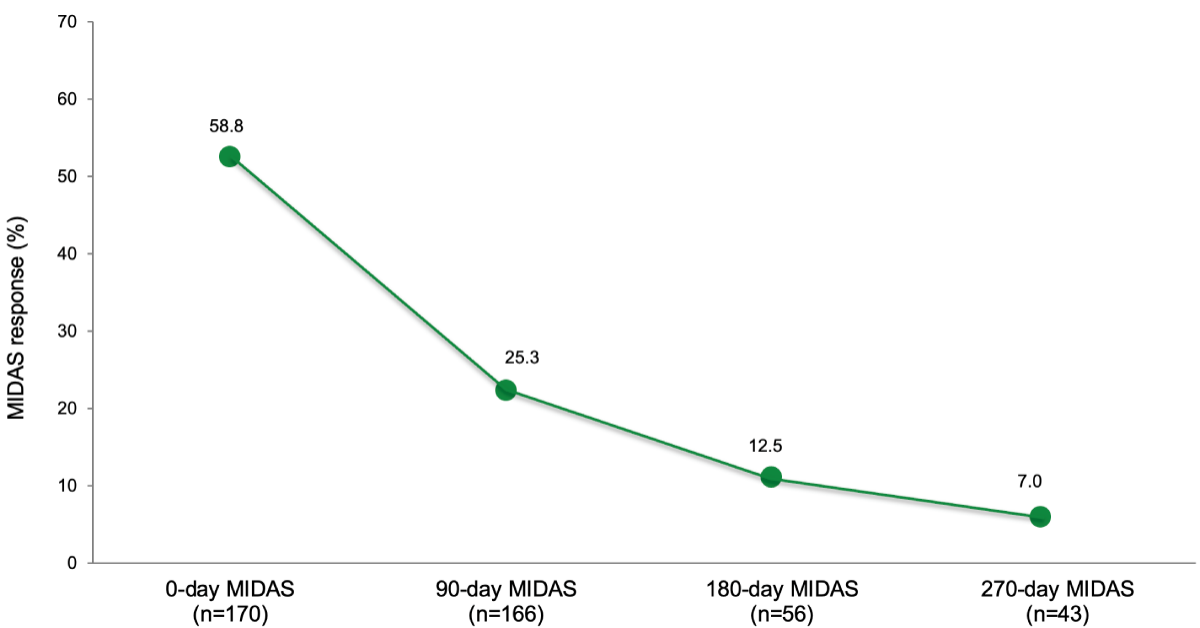
Migraine Disability Assessment Score (MIDAS) response rates and mean scores.

Figure 4 summarizes the responses to the MIDAS questionnaire, with higher scores indicating higher disability. The baseline mean total MIDAS score of responders was 110 (SD 107; range 3-452), with a baseline response rate of 58.8% (100/170). The mean total MIDAS score declined to 71 (SD 65; range 0-260) on day 90 and 78 (SD 57; range 0-180) on day 180. On day 270, the MIDAS response rate declined to 7.5% (3/40).

#### Patient Satisfaction With the Current Treatment Plan

The baseline and subsequent patient satisfaction assessments over the first 180 days are summarized in Table 5. The baseline response rate was 34.5% (57/165). Monthly assessment showed that the response rates ranged from 30.9% (42/136) to 41% (28/68) over the first 6 months. After 6 months, the response rate ranged from 10% (2/20) to 30% (6/20). At baseline, the patients reported that they were very satisfied (10/57, 18%), satisfied (10/57, 18%), neutral (15/57, 26%), dissatisfied (18/57, 32%), and very dissatisfied (4/57, 7%). The percentage of patients reporting that they were satisfied or very satisfied increased to 69% (41/59) at 30 days and ranged from 59% (13/22) to 100% (2/2) thereafter.

**Table 5:** Summary of monthly patient satisfaction with their migraine treatment plan and response rates^a^.

			Baseline (n=57)		Day 30 (n=59)		Day 60 (n=47)		Day 90 (n=42)		Day 120 (n=28)		Day 150 (n=22)		Day 180 (n=18)
**Patient satisfaction, n (%)**															
	Very satisfied	10 (17.5)		12 (20.3)		12 (25.5)		13 (31)		6 (21.4)		5 (22.7)		6 (33.3)	
	Satisfied	10 (17.5)		29 (49.2)		17 (36.2)		15 (35.7)		12 (42.9)		8 (36.4)		9 (50)	
	Neutral	15 (26.3)		15 (25.4)		16 (34)		10 (23.8)		9 (32)		6 (27.3)		3 (16.7)	
	Dissatisfied	18 (31.6)		2 (3.4)		1 (2.1)		3 (7.1)		0 (0)		3 (13.6)		0 (0)	
	Very dissatisfied	4 (7)		1 (1.7)		1 (2.1)		1 (2.4)		1 (3.6)		0 (0)		0 (0)	
Total respondents, n			57		59		47		42		28		22		18
Total very satisfied and satisfied, n (%)			10 (35)		41 (69.5)		19 (61.7)		18 (66.7)		18 (64.3)		13 (59.1)		15 (83.3)
Total questionnaires assigned on this date of enrollment, n			165		158		143		136		68		63		56
Response rate, n (%)			57 (34.5)^b^		59 (37.3)^c^		47 (32.9)^d^		42 (30.9)^e^		28 (41.2)^f^		22 (34.9)^g^		18 (32.1)^h^

^a^Assessments after 180 days were omitted because of the small number of respondents (range 2-8).

^b^n=165.

^c^n=158.

^d^n=143.

^e^n=136.

^f^n=68.

^g^n=63.

^h^n=56.

### Health Care Use Comparison

A total of 121 MICP patients were compared with 62 control patients who received usual care without the MICP. No difference was observed between the 121 MICP intervention patients and controls with respect to age, sex, chronic migraine International Classification of Diseases–10 diagnosis, race, or marital status (Multimedia Appendix 2). No difference was observed in the rate of telephone calls, electronic messages, or emergency department visits between cohorts. Fewer face-to-face visits occurring after MICP enrollment were observed in the MICP arm (13/121, 10.7%) compared with the control group (26/62, 42%; *P*<.001).

## Discussion

### Principal Findings

We observed fever downstream face-to-face clinical visits among participants enrolled in the MICP and no differences in the number of downstream telephone calls, medication refills, or electronic messages between cohorts. The finding that we observed fewer follow-up face-to-face visits in the MICP cohort suggests that the MICP may have provided the health care team with the information needed to modify treatment plans using telephone or electronic communication with patients, rather than relying on information gathered in a formal face-to-face visit. Importantly, we did not observe a shift from face-to-face visits to a higher burden of telephone calls and electronic messages, which remained the same in both the study arms. Reducing unnecessary face-to-face visits for patients whose symptoms can be managed remotely improves access to migraine care for patients with more acute or complex needs. The ability to manage ongoing care remotely without face-to-face visits lowers the cost of care for patients and their insurers and improves the convenience of care and overall access to care. Accountable care organizations may benefit from improvement in access for the sickest patients while providing more efficient care to the population served.

Further study is needed to determine whether patients are more satisfied with remote monitoring and management via telephone or electronic communication compared with traditional face-to-face care requires further study. A prior survey assessment of patient care model preferences in our community migraine population showed that the highest preference was for telephone follow-up regarding medications rather than a face-to-face visit; a visit with a neurologist, especially for those with chronic migraine; and a written action plan in the medical record [14]. In our practice, we already routinely document written action plans in the EHR and use registered nurses to help patients progress through options on the plan if needed. The MICP data viewable in the EHR may facilitate asynchronous electronic care delivery, which is now a reimbursable service [27]. If the MICP were used in primary care, data gathered by primary teams could be used by a neurologist to perform electronic consultations [28], which are also a potential source of revenue.

### MICP Patient Engagement

Our evaluation of patient usability yielded positive results. However, the low sustained engagement and limited reports of patient understanding of self-management education indicate areas that require improvement. Engagement was enhanced by direct health care team to patient electronic communication and automated reminders, which was associated with an increase in patient engagement within 1 to 2 weeks of enrollment. The task completion rates were highest for the first 4 to 5 weeks and then declined with time for all measures. Patients more often answered a question related to joy rather than missed work or impaired productivity, suggesting that the question resonated with the patients. We plan to survey MICP users and aim to better understand how best to improve and sustain patient engagement over time.

Educational materials were delivered accordingly to a predetermined schedule, and we observed that the percentage of patients indicating “I understand” was highest for content delivered early. Further study is needed to determine if this is more a function of the technical user experience than quality, resonance of the education content itself, or the content delivery schedule. It is also unclear if scheduled delivery of education content is preferred over providing a library of content and whether the education content impacts patient engagement with other assigned MICP tasks.

A previously published qualitative study of migraine smartphone app user comments emphasized the importance of a user-friendly design combining the ability to monitor headache features, triggers, and response to treatments with the ability to view trends and export the data [29,30]. The MICP did not include a detailed diary of headache characteristics but did allow patients and providers to view trend data. The MICP did not allow patients to export their medical records. It is not known whether our patients were satisfied with this approach or whether they may have been more engaged if there was the ability to track individual headaches along with response to treatment. Users of the MICP required a diagnosis of migraine by a clinician and did not include an app-based diagnostic algorithm, as previously described [19].

Potential MICP enhancements that may improve engagement include app-based delivery of biofeedback [31], progressive muscle relaxation [32,33], and the ability of patients to write and record free-text observations as they are monitoring [34].

### Strengths

The strengths of this study include the prospective randomized design of the feasibility and downstream use study as well as the size and duration of the study. The strengths of the MICP development process include engagement of PCPs and specialists in the iterative design process. The MICP by design quickly standardized the assessment and monitoring of a large population of patients with migraine evaluated and treated in a community specialty practice. The assessment of usability and observations of headache outcomes may be generalized to other community neurology practices with similar demographics [11].

### Limitations

The limitations of this pilot study include the lack of a prospective comparison of headache outcomes and the fact that it was not designed or powered to study whether patient engagement was associated with headache phenotype, treatment, or response to treatment. Future studies are needed to determine whether MICP improves headache outcomes, including disability and patient satisfaction. As patients were required to tap a specific button within the app to confirm the review of educational materials, we were not able to confirm the time spent reviewing the materials or whether information was reviewed but not recorded. We also did not include patients with migraine during the MICP development process. Further study of usability including assessment of a large population with greater diversity of migraine phenotypes, age, race, ethnicity, and social determinants of health is needed. Finally, further study is needed to better understand the effect of the MICP on downstream care team workload, including telephone calls, electronic massages, and follow-up visits in the outpatient, inpatient, and emergency department settings. We did not deploy the MICP in primary care clinics, where most patients may present for migraine and where the need for education and management support is likely greatest [35]. The MICP was implemented in a unique collaborative neurology primary care practice, which limits the generalizability of the downstream workload observations.

### MICP Improvement Opportunities

On the basis of this initial pilot, we will consider the following changes to the MICP: (1) fewer automatic care team notifications when patients are not engaged with the MICP and instead encourage patients to reengage via automatic electronic messages; (2) develop branching logic to help clarify whether a patient-reported medication concern is about preventive or acute therapy and which pharmacy they prefer for medication refills if needed; (3) education delivered in a single library rather than according to a schedule; (4) use branching logic to push just-in-time self-management education to patients based on headache frequency or medication treatment frequency; (5) the addition of a numeric pain scale to rate headache severity with trends that may be viewed by the patient and provider; and (6) the simplification of the assessments to include branching logic to assess function or disability (missed work, reduced productivity, and reduced joy).

### Future Directions

Future investigations of the MICP intervention will use surveys, interviews, and focus groups to assess patient experiences and inform future interactions with the aim of improving patient engagement over time. We will also implement the MICP in primary care and perform a prospective cohort study or randomized trial on usability, headache outcomes, and downstream use of primary care and neurology care team resources. The MICP has the potential to standardize the assessment and monitoring of patients in primary care, which may facilitate improved ability to increase access and care via electronic consultations [24,28], the use of e-visits [27], or the identification of patients that may benefit most from specialty care [11]. If large populations of patients with migraine were engaged in remote monitoring, population health management strategies could be developed to identify patients in greatest need of limited care resources or proactively offer evidence-based treatment options. Artificial intelligence–based tools hold promise as interventions for electronic headache assessment, treatment preference determination, summarizing past treatment trials for prior authorization purposes, and enhancing communication between patients and the care team while reducing the workload for the care team. Machine learning is already effective for headache diagnosis classification based on patient questionnaires [36-38] and recognition of medication overuse headache [37,39] and could potentially be incorporated into a comprehensive headache assessment and monitoring tool. Using effective rhetoric to promote the adoption of artificial intelligence–based tools is important and may also help improve the engagement of tools such as the MICP in its current design [40]. Data privacy and cybersecurity risks also need to be addressed [41]. Finally, it is important to study the perspectives of clinical care providers and the quadruple aim of improving patient experience, outcomes, and provider experience while reducing the total cost of care [42]. Remote monitoring for chronic health conditions, such as migraine, which are associated with significant health care costs, has the potential to provide access to care without the need for face-to-face visits. Assuming patients have internet access, digital tools may help address disparities in care, particularly for patient populations considered rural and underserved, while minimizing patient burden. The MICP could also be deployed in other populations of patients with access to Epic EHR–supported health care systems that have adopted the MyChart Care Companion functionality.

### Conclusions

mHealth smartphone-based tools may be successfully integrated with the EHR and may facilitate standardized assessment and monitoring of patients with migraine. Usability in a community neurology practice was favorable, although it did not meet our predetermined usability threshold, suggesting opportunities for improvement. The MICP reduced the use of face-to-face visits in our practice while maintaining a similar downstream workload for care teams. Further studies in larger and more diverse populations (race or ethnicity, age, gender, and socioeconomic status) are needed to determine which mobile app design, content, and monitoring features will facilitate sustained high levels of patient engagement and outcomes over time. Further studies in primary care and subspecialty headache clinics are needed to determine the generalizability of our observations.

## Appendix

Multimedia Appendix 1 Summary of the Migraine Interactive Care Plan.

Multimedia Appendix 2 Comparison of demographics and downstream use in Migraine Interactive Care Plan cohort versus controls.

## Abbreviations

EHR: electronic health record
mHealth: mobile health
MICP: Migraine Interactive Care Plan
MIDAS: Migraine Disability Assessment Score
PCP: primary care provider

## Data Availability

All deidentified data sets generated and analyzed during this study are available from the corresponding author upon reasonable request.
