# Supplementary material for: Feasibility and Usability of a Mobile App–Based Interactive Care Plan for Migraine in a Community Neurology Practice: Development and Pilot Implementation Study
Source: JMIR Form Res. 2023 Oct 5;7:e48372. doi: 10.2196/48372 (PMC10587810; doi:10.2196/48372)
Supplement: Multimedia Appendix 1 [file formative_v7i1e48372_app1.pdf]

General Neurology/Primary Care Migraine Delivery Process

8/23/2019

Assessments

Day 1
Migraine management satisfaction
Repeat day 30 and every 30 after until 1 year

Day 1
Migraine Disability Assessment (MIDAS)
Repeat day 90 and every 90 after until 1 year

Day 90
Continue on care plan
Repeat day 90 and every 90 after until 1 year

Weekly

Starting Day 8

Flowsheet
Migraine check-in

Questionnaire
Medication check-in

Education

Day 1 Education
Welcome to the Mayo Clinic Care Plan for Migraine!
1 document

Day 1 Education
Review the facts about migraine headaches
1 document/1 video

Day 2 Education
Understand migraine medications
1 document

Day 4 Education
Know how to treat a migraine attack
1 document

Day 6 Education
Identify your triggers
1 document

Day 8 Education
Count your sleep to prevent migraines
1 document

Day 10 Education
Stay hydrated to stop migraines
1 document

Day 12 Education
Check your eating habits to prevent migraines
1 document

Day 14 Education
Watch your daily routine to avoid migraines
1 document

Day 16 Education
Get moving to avoid migraines
1 document

Day 18 Education
Manage stress for migraine relief
1 document

Day 20 Education
Plan ahead to avoid migraine triggers
1 document

Day 22 Education
Understand complementary therapies for migraine
1 document

Day 24 Education
Women 18-50 only
Learn about menstrual migraines
1 document/1 video

Day 26 Education
*Repeat from Day 4**
Know how to treat a migraine attack
1 document

Day 30 Education
Learn about rebound headaches
1 document

Medication check-in
QNR0001

8/23/2019

QUES0001
In the past week, did you take your migraine medication as prescribed?

Yes | No

QUES0002
Do you have any concerns about your migraine medication?

No Concerns	
I need a medication refill	ESC0016 Notify the care team
QUES0010 Which medication do you need refilled?	FREE TEXT
QUES0011 Please provide the name of your preferred pharmacy for refill, with city and state:	FREE TEXT
Other: Free Text	ESC0013 Notify the care team
QUES0012 Describe your concern:	FREE TEXT
I'm worried about running out of medication	ESC0010 Notify the care team
I'm worried about costs or insurance coverage of medication	ESC0011 Notify the care team
I'm worried about rebound headaches	ESC0014 Education
I'm having side effects from taking medication	ESC0015 Message to patient
I have questions about my migraine medication	ESC0012 Notify the care team

Migraine management satisfaction
QNR002 and QNR003

8/23/2019

QRN002 has no Escalations

QUES0003
How satisfied are you with the plan for managing your migraine?

Response	Escalation
Very satisfied	
Satisfied	
Neutral	
Dissatisfied	ESC0004<br>Notify the care team
Very dissatisfied	ESC0003<br>Notify the care team

Migraine Disability Assessment (MIDAS)
QNR004

8/23/2019

QUES0004<br>On how many days in the last 3 months did you miss work or school because of your headaches?	Value of 0-90,<br>pop up if outside of range
QUES0005<br>How many days in the last 3 months was your productivity at work or school reduced by half or more because of your headaches? (Do not include days counted in question 1 where you missed work or school.)	Value of 0-90,<br>pop up if outside of range
QUES0006<br>On how many days in the last 3 months did you not do household work because of your headaches?	Value of 0-90,<br>pop up if outside of range
QUES0007<br>How many days in the last 3 months was your productivity in household work reduced by half or more because of your headaches? (Do not include days you counted in question 3 where you did not do household work.)	Value of 0-90,<br>pop up if outside of range
QUES0008<br>On how many days in the last 3 months did you miss family, social or leisure activities because of your headaches?	Value of 0-90,<br>pop up if outside of range

Patient inputs from QNR0004 should be totaled and be able to present to provider a total MIDAS Score.

Care plan check-in
QNR005

8/23/2019

QUES0009
Would you like to continue on this care plan and complete weekly check-ins?

Yes	ESC0019<br>Notify care team
No	ESC0009<br>Notify care team
No response after 5 days	ESC0020<br>Notify care team

Migraine check-in
GROUPER as 1 TASK

Used so patients can see progress over time - All to present at 1 time to patient 8/23/2019

FLO0001
In the past week, how many days did you have a headache, even if it was mild? (If headache lasted more than one day, count each day.)

Score 3-7
ESC0001
Weeks 1-6

Score 3-7
ESC0002
Week 7-til end

FLO0002
In the past week, how many days did you treat headache with acute pain medications? (Include over-the-counter and prescription pain relievers.)

FLO0003
In the past week, how many days did you miss work or school because of headaches?

FLO0004
In the past week, how many days was your productivity reduced at work or school because of headaches?

Pop up if not working or school use 0

FLO0005
In the past week, how many days did headaches reduce your joy in social or leisure activities?

Free numeric text
Minimum: 0
Maximum: 7
Pop up if outside of range
