# Supplementary material for: Feasibility and Usability of a Mobile App–Based Interactive Care Plan for Migraine in a Community Neurology Practice: Development and Pilot Implementation Study
Source: JMIR Form Res. 2023 Oct 5;7:e48372. doi: 10.2196/48372 (PMC10587810; doi:10.2196/48372)
Supplement: Multimedia Appendix 2 [file formative_v7i1e48372_app2.docx]

**Multimedia Appendix 2.** Comparison of demographics and downstream use in Migraine Interactive Care Plan (MICP) cohort versus controls.

| Migraine Interactive Care Plan (MICP) versus Control demographics and downstream use | | | MICP (n=121) | Control (n=62) | *P* value |
| --- | --- | --- | --- | --- | --- |
| **Age (years)** | | | | | .88^a^ |
|  | Mean (SD) | | 42.4 (12.2) | 43.3 (14.8) |  |
|  | Median (IQR) | | 42.0 (34.0, 51.0) | 41.5  (30.0, 52.0) |  |
|  | Range | | 20.0-73.0 | 20.0-78.0 |  |
| **Sex, n (%)** | | | | | .89^b^ |
|  | Female | | 110 (90.9) | 56 (90.3) |  |
|  | Male | | 11 (9.1) | 6 (9.7) |  |
| **Chronic migraine ICD-10 diagnosis, n (%)** | | | | | .31^b^ |
|  | No | | 83 (68.6) | 47 (75.8) |  |
|  | Yes | | 38 (31.4) | 15 (24.2) |  |
| **Marital status, n (%)** | | | | | .96^b^ |
|  | Single | | 41 (33.9) | 21 (33.9) |  |
|  | Married | | 65 (53.7) | 32 (51.6) |  |
|  | Divorced | | 11 (9.1) | 7 (11.3) |  |
|  | Widowed | | 1 (0.8) | 1 (1.6) |  |
|  | Life partner | 1 (0.8) | 0 (0.0) |  |  |
|  | Separated | | 2 (1.7) | 1 (1.6) |  |
| **Duration of follow-up after enrollment** | | | | | — |
|  | Mean (SD) | | 169.3 (93.1) | 358.6 (31.1) |  |
|  | Median (IQR) | | 103.0 (95.0, 200.0) | 365.0 (365.0, 365.0) |  |
|  | Range | | 90.0-365.0 | 157.0-365.0 |  |
| **Emergency department visits, n (%)** | | | | | — |
|  | 0 | | 106 (87.6) | 47 (75.8) |  |
|  | 1 | | 12 (9.9) | 10 (16.1) |  |
|  | 2 | | 3 (2.5) | 3 (4.8) |  |
|  | 3 | | 0 (0.0) | 2 (3.2) |  |
| **Emergency room visits per 30 days** | | | | | .09^a^ |
|  | Mean (SD) | | 0.0 (0.1) | 0.0 (0.1) |  |
|  | Median (IQR) | | 0.0 (0.0, 0.0) | 0.0 (0.0, 0.0) |  |
|  | Range | | 0.0-0.6 | 0.0-0.3 |  |
| **Escalation(s) per patient, n (%)** | | | | | — |
|  | 0 | | 5 (4.1) | — |  |
|  | 1 | | 15 (12.4) | — |  |
|  | 2 | | 6 (5.0) | — |  |
|  | 3 | | 8 (6.6) | — |  |
|  | 4 | | 11 (9.1) | — |  |
|  | 5 | | 9 (7.4) | — |  |
|  | 6 | | 7 (5.8) | — |  |
|  | 7 | | 9 (7.4) | — |  |
|  | 8 | | 9 (7.4) | — |  |
|  | 9 | | 5 (4.1) | — |  |
|  | 10 | | 5 (4.1) | — |  |
|  | 11 | | 1 (0.8) | — |  |
|  | 12 | | 2 (1.7) | — |  |
|  | 13 | | 4 (3.3) | — |  |
|  | 14 | | 4 (3.3) | — |  |
|  | 15 | | 1 (0.8) | — |  |
|  | 18 | | 5 (4.1) | — |  |
|  | 21 | | 4 (3.3) | — |  |
|  | 22 | | 1 (0.8) | — |  |
|  | 23 | | 3 (2.5) | — |  |
|  | 24 | | 1 (0.8) | — |  |
|  | 27 | | 1 (0.8) | — |  |
|  | 29 | | 1 (0.8) | — |  |
|  | 33 | | 1 (0.8) | — |  |
|  | 35 | | 1 (0.8) | — |  |
|  | 36 | | 1 (0.8) | — |  |
|  | 40 | | 1 (0.8) | — |  |
| **Electronic in-basket messages, n (%)** | | | | | — |
|  | 0 | | 53 (43.8) | 12 (19.4) |  |
|  | 1 | | 22 (18.2) | 21 (33.9) |  |
|  | 2 | | 11 (9.1) | 11 (17.7) |  |
|  | 3 | | 13 (10.7) | 3 (4.8) |  |
|  | 4 | | 8 (6.6) | 7 (11.3) |  |
|  | 5 | | 5 (4.1) | 1 (1.6) |  |
|  | 6 | | 2 (1.7) | 0 (0.0) |  |
|  | 7 | | 2 (1.7) | 2 (3.2) |  |
|  | 8 | | 2 (1.7) | 2 (3.2) |  |
|  | 9 | | 1 (0.8) | 1 (1.6) |  |
|  | 10 | | 1 (0.8) | 1 (1.6) |  |
|  | 11 | | 1 (0.8) | 0 (0.0) |  |
|  | 12 | | 0 (0.0) | 1 (1.6) |  |
| **Electronic in-basket messages per 30 days** | | | | | .96^a^ |
|  | Mean (SD) | | 0.4 (0.5) | 0.2 (0.2) |  |
|  | Median (IQR) | | 0.2 (0.0, 0.5) | 0.1 (0.1, 0.3) |  |
|  | Range | | 0.0-2.9 | 0.0-1.0 |  |
| **Telephone calls, n (%)** | | | | | — |
|  | 0 | | 85 (70.2) | 37 (59.7) |  |
|  | 1 | | 25 (20.7) | 10 (16.1) |  |
|  | 2 | | 5 (5.0) | 6 (9.7) |  |
|  | 3 | | 4 (2.5) | 3 (4.8) |  |
|  | 4 | | 1 (0.8) | 4 (6.5) |  |
|  | 5 | | 0 (0.0) | 1 (1.6) |  |
|  | 6 | | 0 (0.0) | 1 (1.6) |  |
|  | 11 | | 1 (0.8) | 0 (0.0) |  |
| **Telephone calls per 30 days** | | | | | .43^a^ |
|  | Mean (SD) | | 0.1 (0.2) | 0.1 (0.1) |  |
|  | Median (IQR) | | 0.0 (0.0, 0.2) | 0.0 (0.0, 0.1) |  |
|  | Range | | 0.0-1.8 | 0.0-0.5 |  |
| **Clinic visits after enrollment, n (%)** | | | | | — |
|  | 0 | | 108 (87.6) | 36 (58.1) |  |
|  | 1 | | 9 (7.4) | 24 (38.7) |  |
|  | 2 | | 1 (0.8) | 1 (1.6) |  |
|  | 3 | | 1 (0.8) | 1 (1.6) |  |
|  | 4 | | 2 (1.7) | 0 (0.0) |  |
| **Clinic visits per 30 days** | | | | | <.001^a^ |
|  | Mean (SD) | | 0.0 (0.1) | 0.0 (0.1) |  |
|  | Median (IQR) | | 0.0 (0.0, 0.0) | 0.0 (0.0, 0.1) |  |
|  | Range | | 0.0-1.0 | 0.0-0.3 |  |
| **Medication refill, n (%)** | | | | | — |
|  | 0 | | 106 (87.6) | 56 (90.3) |  |
|  | 1 | | 2 (1.7) | 2 (3.2) |  |
|  | 2 | | 6 (6.6) | 4 (6.5) |  |
|  | 3 | | 1 (0.8) | 0 (0.0) |  |
|  | 4 | | 2 (1.7) | 0 (0.0) |  |
|  | 5 | | 1 (0.8) | 0 (0.0) |  |
|  | 8 | | 1 (0.8) | 0 (0.0) |  |
| **Medication refill per 30 days** | | | | | .44^a^ |
|  | Mean (SD) | | 0.1 (0.3) | 0.0 (0.0) |  |
|  | Median (IQR) | | 0.0 (0.0, 0.0) | 0.0 (0.0, 0.0) |  |
|  | Range | | 0.0-1.9 | 0.0-0.2 |  |

^a^Wilcoxon rank-sum test.

^b^Chi-square test.

- summary data only; see comparison of rate of event/ 30 days
